# THE INFLUENCE OF ANXIETY AND PERCEIVED CONTROL OF ANXIETY ON OLDER VETERANS’ QUALITY OF LIFE

**DOI:** 10.1093/geroni/igae098.2680

**Published:** 2024-12-31

**Authors:** Carter Davis, Chalise Carlson, Sherry Beaudreau, Christine Gould

**Affiliations:** VA Palo Alto Healthcare System, Palo Alto, California, United States; Department of Veterans Affairs, Palo Alto, California, United States; VA Palo Alto Healthcare System, Palo Alto, California, United States; VA Palo Alto Healthcare System, Palo Alto, California, United States

## Abstract

Anxiety disorders are prevalent among older military Veterans, and the presence of an anxiety disorder negatively impacts quality of life. Little is understood, however, about potential psychological processes that explain how quality of life is impacted by anxiety among older Veterans. One potential mechanism is anxiety control, or one’s perceived ability to manage their anxiety as it arises in various contexts. In this study, baseline data were analyzed from 62 older Veterans (M age = 71.2) who participated in a clinical trial of a guided anxiety management intervention. The most common diagnoses among participants were anxiety not otherwise specified (37.1%), and generalized anxiety disorder (22.6%). Linear regression models were created which tested anxiety symptom severity as a predictor of mental health-related quality of life, with anxiety control as a covariate. Lower anxiety severity (p <.001) and higher perceived control of anxiety (p =.013) were found to be significant predictors of quality of life. Anxiety control remained a significant predictor (p =.05) when anxiety diagnosis type was included as a covariate, but not when depression symptom severity and health comorbidity were included in the model (p =.19). These results indicate anxiety control as a potentially important target in interventions for older adult Veterans across anxiety disorder subtypes. However, larger samples are needed to clarify the path through which this effect operates and the influence of additional factors such as depression and comorbid health conditions, which could be examined through moderation and mediation analysis.

